# Carbohydrate knowledge, dietary guideline awareness, motivations and beliefs underlying low-carbohydrate dietary behaviours

**DOI:** 10.1038/s41598-020-70905-2

**Published:** 2020-09-02

**Authors:** Chaitong Churuangsuk, Michael E. J. Lean, Emilie Combet

**Affiliations:** Human Nutrition, School of Medicine, Dentistry, and Nursing, College of Medical, Veterinary and Life Sciences, University of Glasgow, Room 2.22, Level 2, New Lister Building, 10-16 Alexandra Parade, Glasgow Royal Infirmary, Glasgow, G31 2ER UK

**Keywords:** Nutrition, Obesity, Type 2 diabetes

## Abstract

To explore the factors (including knowledge and attitude) influencing the decision to follow a low-carbohydrate diet (LCD) or not in a sample of the UK population. An online questionnaire was distributed electronically to adults who had either followed LCD or not (February–December 2019). Demographics and self-reported “LCD-status” (current, past and non-follower) were collected. Multivariable linear regression was used with carbohydrate knowledge, dietary guideline agreement and theory of planned behaviour (TPB) constructs (all as predictors) to explain the intention to follow a LCD (outcome). Respondents (n = 723, 71% women, median age 34; 85% white-ethnicity) were either following (n = 170, 24%) or had tried a LCD in the preceding 3 months (n = 184, 25%). Current followers had lower carbohydrate knowledge scores (1–2 point difference, scale − 11 to 11) than past and non-followers. A majority of current LCD followers disagreed with the EatWell guide recommendations *“Base meals on potatoes, bread, rice and pasta, or other starchy carbohydrates. Choose whole grains where possible”* (84%) and *“Choose unsaturated oils and spreads and eat in small amounts such as vegetable, rapeseed, olive and sunflower oils”* (68%) compared to past (37%, 10%, respectively) and non-followers (16%, 8%, respectively). Weight-loss ranked first as a motivation, and the internet was the most influencial source of information about LCDs. Among LCD-followers, 71% reported ≥ 5% weight loss, and over 80% did not inform their doctor, nurse, or dietitian about following a diet. Approximately half of LCD followers incorporated supplements to their diets (10% used multivitamin/mineral supplements), despite the restrictive nature of the diet. TPB constructs, carbohydrate knowledge, and guideline agreement explained 60% of the variance for the intention to follow a LCD. Attitude (std-β = 0.60), perceived behavioural control (std-β = 0.24) and subjective norm (std-β = 0.14) were positively associated with the intention to follow a LCD, while higher knowledge of carbohydrate, and agreeing with national dietary guidelines were both inversely associated (std-β = − 0.09 and − 0.13). The strongest primary reason behind UK adults’ following a LCD is to lose weight, facilitated by attitude, perceived behavioural control and subjective norm. Higher knowledge about carbohydrate and agreement with dietary guidelines are found among people who do not follow LCDs.

## Introduction

Low-carbohydrate and reduced carbohydrate diets (LCD) have become popular for weight loss and as a possible strategy for type 2 diabetes management^1^. Although not superior to conventional weight-loss diets, LCD interventions can generate weight loss, and improve glucose control in people with obesity and/or diabetes^2, 3^. LCDs have been used intermittently for centuries but gained recent popularity ever since the Atkins diet was first introduced in 1972^4^. Successful weight loss journeys with LCDs by celebrities have been highlighted in the media, partly driving the renewed appeal of LCDs for the public^5, 6^. Media polls have estimated that 3 million people in the UK, approximately 7% of men and 10% of women, have tried LCDs^7^, similar to 7% in a population survey in Finland^8^, and up to 17% in surveys from the US^9^ and Australia^10^. A recent Food and Health survey^11^ of 1,009 Americans in 2018 also showed that 16% of respondents had followed a LCD, similar to findings in 2008^9^.

There is a lack of a consensus what constitutes a diet “low” in carbohydrates, with references to carbohydrate-reduced diets, low-carbohydrate diets (LCDs), low-carbohydrate high-fat diets (LCHF), creating a major issue when communicating about these diets. A group of experts in the field has recommended that a carbohydrate content < 26% of energy (or < 130 g/day) could qualify as a criterion for LCDs^12^. However, our recent systematic review of published meta-analyses of LCDs found that the criteria for LCDs in the literature varied significantly^2^. Some authors defined LCDs based on a maximum intake of carbohydrate from 60 g per day^13–16^, to a maximum of 120 g per day^17^ without energy restriction (most trials of LCDs used ad libitum recommendation), while other authors defined LCDs based on maximum percentage energy contribution from carbohydrate ranging from 20% of energy^13, 15^, to 45% of energy per day^18–20^. With this range of criteria and definitions, understanding of what constitutes a LCD in the lay population is likely to vary, and are likely to include diets such as the Atkins diet, ketogenic diet or Paleolithic diet.

Despite the increasing popularity of LCDs, little is known about the nutritional behaviour of people who identify as LCD followers, including their socio-economic backgrounds^21^. Analysis of the possible impacts on health may be confounded by reverse causality in surveys, as these individuals may have pre-existing illness, and many other determinants could influence a decision to try to follow, or not follow, a particular diet^22^. Poor or incomplete knowledge on the nutritional compositions of foods and the balance of an overall diet, coupled with incorrect beliefs about food and health, may put people who attempt to follow a LCD at risk if their diet is not guided professionally^1, 23, 24^. National dietary guidelines are compiled by independent scientific and medical experts, based on the totality of the nutritional evidence, and there is evidence that higher adherence to dietary guidelines is linked with a reduction in chronic disease risks^25–27^. Knowledge of guideline recommendations is intended to improve individuals’ food choices, but journalistic scepticism and a lack of understanding of scientific evidence can lead to inappropriate rejection of guidelines in many fields^28, 29^.

Among the theoretical models/theories commonly used to explain human behaviours, in this case food choice, the *Theory of Planned Behaviour (TPB)* is widely accepted as valid and has been extensively used to study dietary behaviour and to design interventions for behavioural change^30–32^. The other popular model is the transtheoretical model of behaviour change (TMBC), which is used to identify people’s stage of change and to tailor interventions to changes in their behaviour. The rationale for including the TPB over the TMBC is that the aim of this paper is to explore the factors influencing following a LCD (or not), with the TPB specifically identifying intention as a central concept and exploring explicit factors explaining the intention to perform the chosen behaviour^33^. The TPB model identifies *intention* as the most powerful psychological determinant to eat a LCD or not, predicted by three other psychological constructs: *attitude towards the LCD behaviour* (i.e. the perception of benefits or harms of following a LCD), *subjective norm* (i.e. the social pressure or the perception of others would want one to follow or not to follow a LCD), and *perceived behavioural control* over the behaviour (i.e. the perception of self-efficacy or ability, or the perception of ease or difficulty to follow a LCD)^30, 31^.

Few studies have explored dietary intake or motivations in self-reported LCD followers^8, 21, 34^, and the empirical evidence is lacking to explain low-carbohydrate dieting behaviour using the TPB theoretical framework. Furthermore, the perceptions or opinions regarding LCDs among people who are not following this diet are not documented. Therefore, the present study investigates the possible factors which might help understand decisions to follow a LCD, or not, in UK adults.

## Results

### Respondents’ characteristics

A total of 723 respondents (515 women, 71%) completed the online questionnaire, with a median age of 34 years and 85% identifying as ‘white’ ethnicity (Table 1). Nearly half of all respondents were either following (n = 170) or had previously tried any LCDs (n = 184). Median body mass index (BMI) of current LCD followers (25.5 kg/m^2^) and past followers (26.4 kg/m^2^) were higher than non-followers (23.7 kg/m^2^, *p* < 0.001; Table 1). Current and past LCD followers also had a higher number of co-morbidities than non-followers. Type 2 diabetes and dyslipidaemia were more prevalent in current followers compared to past and non-followers (Table 1).

**Table 1:** Socio-demographic data (n, %).

	All	Current followers	Past followers	Non-followers	P-value^1, 2^
**No. of respondents**	723	170	184	*369*	
Sex: female	515 (71)	108 (64)^a^	154 (84)^b^	253 (69)^a^	< 0.001
Age, median (IQR)	34 (26–48)	47 (35–56)^a^	35 (27–45)^b^	30 (24–41)^b^	< 0.001
BMI, median (IQR)	24.6 (21.8–28.8)	25.5 (23.1–30.1)^a^	26.4 (22.8–32.8)^a^	23.7 (21.2–27.2)^b^	< 0.001
Ethnicity: white	615 (85)	148 (87)	160 (87)	307 (83)	0.36
**Annual income (GBP)**					< 0.001
No income	28 (4)	3 (2)	6 (3)	19 (5)	
< £15,000	101 (14)	19 (11)	21 (11)	61 (17)	
£15,001—£30,000	165 (23)	25 (15)^a^	53 (29)^b^	87 (24)^b^	
£30,001—£50,000	177 (25)	46 (27)	44 (24)	87 (24)	
£50,001—£80,000	106 (15)	33 (19)^a^	32 (17)^a, b^	41 (11)^b^	
> £80,000	48 (7)	21 (12)^a^	11 (6)^a, b^	16 (4)^b^	
Prefer not to say	98 (14)	23 (14)	17 (9)	58 (16)	
**Education level**					0.23
< Bachelor	264 (37)	47 (28)	73 (40)	144 (39)	
Bachelor	215 (30)	58 (34)	55 (30)	102 (28)	
MSc/PhD/postgrad	229 (32)	61 (36)	53 (29)	115 (31)	
prefer not to say	15 (2)	4 (2)	3 (2)	8 (2)	
**Study field**					0.01
Nutrition and dietetics	58 (8)	9 (5)	12 (7)	37 (10)	
Food science	9 (1)	0	2 (1)	7 (2)	
Medicine	38 (5)	16 (9)^a^	8 (4)^a, b^	14 (4)^b^	
Other HCPs	59 (8)	20 (12)	16 (9)	23 (6)	
Not related to nutrition and health	559 (77)	125 (74)	146 (79)	288 (78)	
**No. of supplement use**					< 0.001
None	446 (62)	86 (51)^a^	115 (63)^a,b^	245 (66)^b^	
1	142 (20)	29 (17)	38 (21)	75 (20)	
2	61 (8)	21 (12)^a^	17 (9)^a, b^	23 (6)^b^	
≥ 3	74 (10)	34 (20)^a^	14 (8)^b^	26 (7)^b^	
**Supplement use**					
Multivitamins/minerals	62 (9)	16 (9)	18 (10)	28 (8)	0.62
Vitamin B complex	11 (2)	4 (2)	2 (1)	5 (1)	0.58
Folate	10 (1)	6 (4)^a^	2 (1)^a, b^	2 (0.5)^b^	*0.02*
Vitamin B12	24 (3)	6 (4)	9 (5)	9 (2)	0.31
Vitamin C	44 (6)	15 (9)	10 (5)	19 (5)	0.23
Vitamin D	93 (13)	37 (22)^a^	22 (12)^b^	34 (9)^b^	< 0.001
Calcium	11 (2)	6 (4)^a^	0^b^	5 (1)^a, b^	*0.024*
Magnesium	40 (6)	29 (17)^a^	1 (0.5)^b^	10 (3)^b^	< *0.001*
Iron	22 (3)	2 (1)	7 (4)	13 (4)	0.27
Zinc	20 (3)	10 (6)^a^	4 (2.2)^a, b^	6 (1.6)^b^	*0.017*
**Smoking status**					< 0.001
Current smokers	66 (9)	5 (3)^a^	21 (12)^b^	40 (11)^b^	
Ex-smokers	107 (15)	37 (22)^a^	34 (19)^a^	36 (10)^b^	
None	545 (76)	128 (75)^a, b^	127 (70)^b^	290 (79)^a^	
**No. of co-morbidities**					< 0.001
None	472 (65)	87 (51)^a^	109 (59)^a^	276 (75)^b^	
1	171 (24)	49 (29)	47 (26)	75 (20)	
2	58 (8)	23 (14)^a^	20 (11)^a^	15 (4)^b^	
≥ 3	22 (3)	11 (7)^a^	8 (4)^a^	3 (1)^b^	
**Comorbidities**					
Type 2 diabetes	32 (4)	22 (13)^a^	9 (5)^b^	1 (0.3)^c^	< 0.001
Dyslipidaemia	38 (5)	22 (13)^a^	8 (4)^b^	8 (2)^b^	< 0.001
Hypertension	60 (8)	26 (15)^a^	19 (10)^a^	15 (4)^b^	< 0.001
Obesity (BMI ≥ 30 kg/m^2^)	156 (22)	43 (25)^a^	62 (34)^a^	51 (14)^b^	< 0.001
**Physical activity**^**3**^					0.019
Fairly active	200 (28)	34 (20)^a^	62 (34)^b^	104 (28)^a, b^	
Moderately active	307 (43)	71 (42)	74 (40)	162 (44)	
Very active	216 (30)	65 (38)^a^	48 (26)^b^	103 (28)^b^	
**Main cook in house**	620 (86)	147 (88)	161 (88)	312 (85)	0.460
Perceived confidence in cooking abilities, mean (SD)^4,^^5^	5.7 (1.3)	6.2 (1.0)	5.6 (1.3)	5.4 (1.3)	< 0.001

Values are n (%) unless otherwise indicated.

^1^Chi-squared test with Bonferroni adjustment for column proportion comparison (categorical data)—different letters represent significant differences between groups.

^2^Kruskal–Wallis Test with Bonferroni correction for multiple tests (continuous data)—different letters represent significant differences between groups.

^3^Fairly inactive—walking only; moderately active—occasionally take exercise, that raise my heart rate, less than 3 times per week; very active—regularly take exercise, that raise my heart rate, 3 times a week or more.

^4^Range 1–7: least—highest.

^5^ANOVA with post-hoc Bonferroni comparison.

Nearly 60% of current LCD followers reported an annual household income level of at least £30,000 (UK) per year (approximately $35,000 US), compared to 47% of past followers and 39% of non-followers (Table 1). There was no difference in education level among LCD followers and non-followers, while confidence in cooking abilities was higher in current LCD followers (Table 1). Interestingly, those with a study background in medicine were represented in a greater proportion among current LCD followers (9%) than past (4%) or non-followers (4%; *p* = 0.01; Table 1).

### Nutritional supplement use

Nearly half of current LCD followers took nutritional supplements, compared to 37% of past followers and 34% of non-followers (*p* < 0.001; Table 1). Among them, 20% of current LCD followers took 3 or more supplements, double the proportion of past (8%) and non-followers (7%) (Table 1). Vitamin D was the most used supplement overall (13% of study respondents), highest in current LCD followers (22%), compared to past (12%) and non-followers (9%; *p* < 0.001; Table 1). Folate (4%), calcium (4%), magnesium (17%) and zinc (6%) were all used more often by current LCD followers than past and non-followers (all* p* < *0.05*; Table 1). Approximately 10% across LCD-followers and non-followers used multivitamins/minerals supplements.

### Frequency of consumption and estimation of foods and nutrient intake as assessed by Dietary Targets Monitor (DTM)

Dietary assessment using the DTM food frequency questionnaire enabled monitoring of dietary targets, rather than assessing actual intakes. The tool allowed a comparison of the estimated intake and frequency of consumption between groups of respondents. Consumption of starchy foods (breads, cereals, pasta, rice, and potatoes excluding chips) was low (median intake of 0.9 times/week; IQR 0.5–4.5) in current LCD followers compared to past followers (12 times/week; IQR 7.6–16.5) and non-LCD followers (14.5 times/week; IQR 10–20; *p* < 0.001; Table 2).

**Table 2:** Food intake as assessed by the dietary targets monitor (DTM).

	Current followers		Past followers		Non-followers		P-value^4^
	median	IQR	median	IQR	median	IQR	
**Median frequency of consumption (times/week) with interquartile range**							
Starchy foods^1^	0.88^a^	0.50–4.50	12.00^b^	7.60–16.50	14.50^c^	10.00–20.00	< 0.001
Breads	0.13^a^	0.13–0.50	3.00^b^	3.00–7.00	7.00^c^	3.00–7.00	< 0.001
Breakfast cereals	0.13^a^	0.13–0.50	3.00^b^	0.50–7.00	3.00^b^	0.50–7.00	< 0.001
Potatoes (other than chips, crisps)	0.13^a^	0.13–0.50	1.00^b^	1.00–3.00	3.00^b^	1.00–3.00	< 0.001
Pasta, rice	0.13^a^	0.13–0.50	3.00^b^	1.00–3.00	3.00^c^	1.00–3.00	< 0.001
Chips	0.13^a^	0.13–0.50	0.50^b^	0.50–1.00	1.00^b^	0.50–3.00	< 0.001
Meat (beef, pork, lamb)	7.00^a^	3.00–7.00	3.00^b^	0.50–5.50	3.00^b^	0.50–5.50	< 0.001
Processed meat (sausage, ham)	1.00^a^	0.50–3.00	1.00^b^	0.13–3.00	1.00^b^	0.13–3.00	0.001
Cheese	5.50^a^	3.00–7.00	3.00^b^	1.00–3.00	3.00^b^	1.00–5.50	< 0.001
Chicken	3.00	1.00–5.50	3.00	1.00–5.50	3.00	1.00–3.00	0.076
Fish^2^	3.13^a^	1.40–6.00	1.13^b^	0.63–2.00	1.00^b^	0.25–2.00	< 0.001
White fish	1.00^a^	0.50–3.00	0.50^b^	0.13–1.00	0.50^b^	0.13–1.00	< 0.001
Oily fish	1.00^a^	0.50–3.00	0.50^b^	0.13–1.00	0.50^b^	0.13–1.00	< 0.001
Fruit and vegetables^3^	22.25^a^	14.20–38.50	32.50^b^	19.50–49.00	28.00^b^	16.75–49.00	< 0.001
Fruits	3.00^a^	0.50–7.00	7.00^b^	3.00–17.50	7.00^b^	5.50–17.50	< 0.001
Vegetables	18.63	11.10–31.50	18.00	11.50–31.50	19.50	10.00 -31.50	0.490
Beans and pulse	3.00	0.50–5.50	3.00	1.00–5.50	3.00	0.50–3.00	0.094
Sweet, chocolates	0.13^a^	0.13–1.00	3.00^b^	1.00–5.50	3.00^b^	1.00–7.00	< 0.001
Ice cream	0.13^a^	0.13–0.50	0.50^b^	0.13–1.00	0.50^b^	0.13–1.00	< 0.001
Crisps, savoury snacks	0.13^a^	0.13–0.50	3.00^b^	0.50–3.00	3.00^b^	0.50–5.50	< 0.001
Cake, scones, sweet pies, or pastries	0.13^a^	0.13–0.50	1.00^b^	0.50–3.00	1.00^b^	0.50–3.00	< 0.001
Biscuit	0.13^a^	0.13–0.50	1.00^b^	0.50–3.00	1.00^b^	0.50–3.00	< 0.001
Fruit juice (not squash)	0.13^a^	0.13–0.13	0.50^b^	0.13–1.00	0.50^b^	0.13–3.00	< 0.001
Soft drinks	0.13^a^	0.13–0.13	0.13^b^	0.13–0.50	0.50^c^	0.13–1.00	< 0.001
Sugar free soft drinks	0.13^a^	0.13–1.00	0.50^b^	0.13–3.00	0.50^b^	0.13–3.00	0.001
**Estimation of key foods and nutrient intake**							
Fruit and vegetables (g/day)	338^a^	216–585	494^b^	296–745	425^b^	255–745	< 0.001
Fish (g/week)	371^a^	167–713	134^b^	74–238	119^b^	30–238	< 0.001
Total fat (g/day)	69.4	51.7–99.5	64.6	41.3–88.7	68.8	46–101.7	0.22
Saturated fat (g/day)	34.7^a^	24.7–51.1	29.5^b^	18.7–41.4	31.6^a,b^	21.2–46	0.024
**Percentage of meeting targets**	**N**	**%**	**N**	**%**	**N**	**%**	**P-value** ^**5**^
Fruit and vegetables (≥ 400 g/day)	72	42%	114	62%	221	70%	0.001
Fish (≥ 360 g/week)	88	52%	40	22%	73	20%	< 0.001

^1^Starchy food was the sum of breads, cereals, potatoes excluding chips, and pasta and rice.

^2^Fish was the sum of white fish and oily fish.

^3^Fruit and vegetable was the sum of fruits and vegetables.

^4^Kruskal–Wallis Test with the Bonferroni correction for multiple tests—different letters represent significant differences between groups.

^5^Chi-square test.

The DTM was not designed to calculate absolute intake of starchy foods, so we assumed that a strict LCD would limit starchy foods to ≤ 1 time/day. With this assumption, 81% of current LCD followers strictly consumed starchy foods ≤ 1 time/day. Approximately 20% of past-LCD followers still limited their starchy food intake to ≤ 1 time/day. Interestingly, 10% of non-LCD followers also reported starchy foods consumption of ≤ 1 time/day. Considering other carbohydrate-rich foods (fruits, sweets and chocolates, ice cream, crisps and snacks, cake and pastries, biscuit, fruit juice, and soft drinks) in combination to starchy foods intake, we found that 48% of current LCD followers strictly limited all carbohydrate-rich foods intake to ≤ 1 time/day.

In contrast, consumption of meat and processed meat, cheese, and fish were greater in current LCD followers than past and non-followers. There was no difference in the frequency of chicken, beans and pulse intake between LCD followers and non-followers (median intakes of 3 times/week, Table 2). Current LCD followers reported consumption of sweet, chocolates, ice cream, snacks, cake and pastries, biscuit and soft drinks (0.13 times/week across all food items) lower than past and non-followers (consumption ranging from 0.13 to 3 times/week, Table 2).

The DTM estimates the absolute intakes of fish, fruit and vegetables and fat, in order to evaluate whether they met the dietary targets. Greater amounts of fish were consumed by current LCD followers (median 371 g/week) compared to past followers (134 g/week) and non-followers (119 g/week) (Table 2). Approximately half of current LCD followers met the target for total fish intake, compared to 20% of past and non-followers (*p* < 0.001; Table 2).

Fruits and vegetables intakes were also lower in current LCD followers (median 338 g/day), compared to 494 g/day and 425 g/day in past followers and non-followers (*p* < 0.001). Similarly, 42% of current LCD followers met the target for fruits and vegetables intakes (≥ 400 g/day), which was lower than past (62%) and non-followers (70%; *p* = 0.001; Table 2). When looking at the frequency of consumption, we found that fruit was consumed less by current followers than past and non-followers, while there was no difference in vegetable consumption (Table 2). Consumption of saturated fat was higher in current followers (median 35 g/day; IQR 25–51) than past followers (median 30 g/day; IQR 19–41), while there was no difference in total fat intake across all groups (median intake ranged from 65 to 69 g/day, p = 0.22).

### Perceived understanding and knowledge of carbohydrates

When asking about perceived understanding of carbohydrates, 89% of current LCD followers reported that they had a very good idea about carbohydrates, higher than past (59%) and non-LCD followers (47%; Table 3). However, the median carbohydrate knowledge score of current LCD followers (6; IQR 5–8) was lower than scores of past (8; IQR 6–9) and non-LCD followers (7; IQR 5–9; Table 3).

**Table 3:** Knowledge and perceived understanding of carbohydrates, and dietary guidelines agreement and awareness stratified by self-reported low-carbohydrate diet status.

	All (n 723)	Current followers (n 170)	Past followers (n 184)	Non-followers (n 369)	P-value
**Perceived understanding, n (%)**					< 0.001^1^
Yes, very good idea	434 (60)	152 (89)^a^	109 (59)^b^	173 (47)^c^	
Yes, vague idea	219 (30)	15 (9)^a^	63 (34)^b^	141 (38)^b^	
Not really	61 (8)	3 (2)^a^	12 (7)^a,b^	46 (13)^b^	
Not at all	9 (1)	0^a^	0^a^	9 (2)^a^	
**Carbohydrate knowledge score, median (IQR)**	7 (5, 9)	6 (5, 8)^a^	8 (6, 9)^b^	7 (5, 9)^b^	< 0.001^2^
**Quartiles of carbohydrate knowledge score, n (%)**^**3**^					< 0.001^1^
Quartile 4	19 (17)	14 (8)	39 (21)	69 (19)	
Quartile 3	198 (27)	33 (19)	46 (25)	102 (28)	
Quartile 2	189 (26)	55 (32)	63 (34)	88 (24)	
Quartile 1	217 (30)	68 (40)	36 (20)	110 (30)	
**Agreement with the UK Eatwell guide, median (IQR)**	2 (0, 4)	− 2 (− 4, 1)^a^	2 (1, 4)^b^	3 (2, 4)^c^	< 0.001^2^
**Eatwell guide, n (%)**					
Aware of	348 (48)	113 (67)	88 (48)	147 (40)	
Follows	87 (12)	8 (5)	23 (13)	56 (15)	
**My plate, n (%)**					
Aware of	79 (11)	47 (28)	13 (7)	19 (5)	
Follows	3 (0.4)	–	–	3 (0.8)	

^1^Chi-squared test with Bonferroni adjustment for column proportion comparison.

^2^Kruskal–Wallis Test with Bonferroni correction for multiple tests—different letters represent significant differences between groups.

^3^Quartiles of knowledge score: Quartile 4 (highest quartile, score bracket 10–11); Quartile 3 (score bracket 8–9); Quartile 2 (score bracket 6–7); Quartile 1 (lowest quartile, score bracket − 11 to 5).

We further explored whether the level of perceived understanding of carbohydrates aligned with knowledge. Among past LCD followers and non-followers, respondents who had higher confidence about their understanding of carbohydrates also had higher knowledge score (r = 0.27 and 0.37, *p* < 0.001). This correlation was not evident among current LCD followers (r = − 0.05, p = 0.54), with knowledge scores similar across levels of perceived understanding (Supplemental Figure S1). Notably, although 89% of current followers reported ‘very good’ idea about carbohydrates, only 28% (n = 47) of them achieved an above-median knowledge score (Table 3).

### Agreement with dietary guidelines

Approximately half of the study sample was aware of existing national dietary guidelines (Table 3). Current LCD followers were more aware of the Eatwell guide (67%) than past (48%) and non-followers (40%). A minority (5%) of current LCD followers reported following the guideline, 3-time less than past and non-followers (13% and 15%; Table 3).

**Figure 1:**
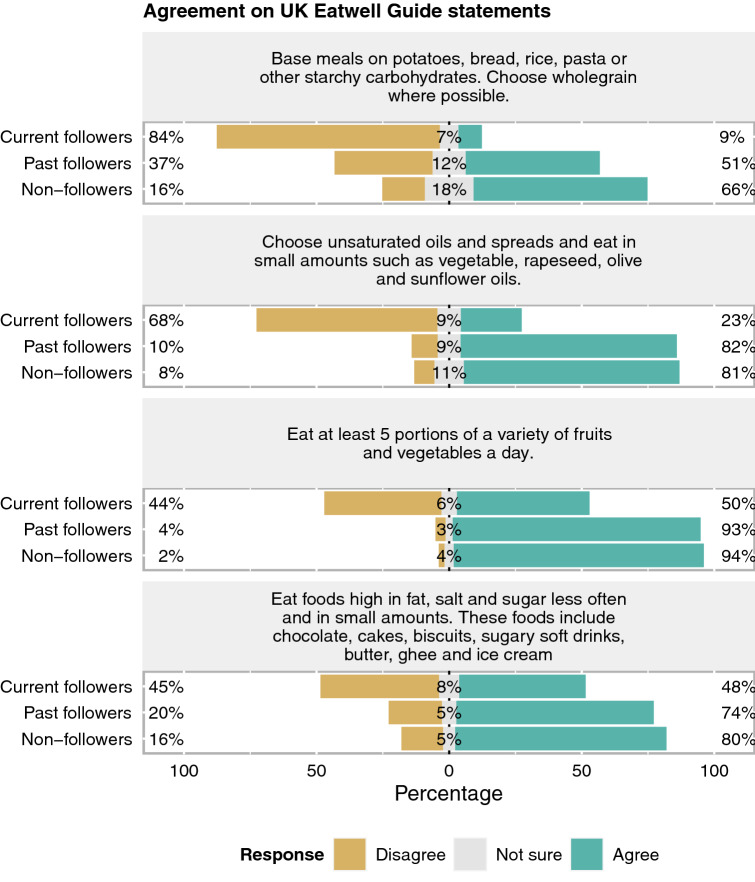
Percentages of participants who agreed on the UK Eatwell Guide statements stratified by low-carbohydrate diet status (n = 723).

Main disagreement of current LCD followers with individual UK Eatwell guide statements, focused on *‘base meals on potatoes, bread, rice, pasta or other starchy carbohydrates. Choose wholegrain where possible’* (84%), followed *‘choose unsaturated oils and spreads and eat in small amounts such as vegetable, rapeseed, olive and sunflower oils’* (68%). On the other hand, a majority of past and non-followers agreed with the statement about vegetable oils (82% and 81%), 5-a-day fruit and vegetable intake (93% and 94%) and recommendation to eat less foods high in fat, salt and sugars (e.g. cake, biscuit, soft drinks) (74% and 80%). The statement about starchy foods was the most divergent finding between groups (Fig. 1).

### Nutritional behaviour of current and past LCD followers

The most common type of LCD followed (past and current) were individual variations on the concept, not aligned with any specific named diet (57%), followed by the ketogenic diet (40%), and the Atkins diet (27%). The median of duration following the diet was 8 months (Table 4). Top three motivations to follow a LCD included losing weight (59%), perception of LCDs being better for health (22%), and for diabetes management (10%) (Table 4). Most respondents consumed a LCD every day at every meal (48%), 4–5 days a week (32%), weekday only (10%) and up to 3 days a week (6%). Nearly 60% reported that the portion size of their meals was smaller while following a LCD, while 22% reported bigger portion sizes (Table 4).

**Table 4:** Dietary behaviour of current and past low-carbohydrate diet followers (n, %).

	All (n 354)	Current (n 170)	Past (n 184)	P-value^5,6^
**Types of LCD**				
No specific name/own variations	203 (57)	112 (66)	91 (50)	0.002
Ketogenic diet	140 (40)	93 (55)	47 (26)	< 0.001
Atkins diet	97 (27)	37 (22)	60 (33)	0.02
Gluten free diet with and without LCD	61 (17)	36 (21)	25 (14)	0.06
Paleolithic diet	50 (14)	31 (18)	19 (10)	0.03
**Source of information/inspiration**				
Internet	97 (27)	55 (32)	42 (23)	0.045
HCPs	56 (16)	28 (17)	28 (15)	0.75
Social media	50 (14)	29 (17)	21 (11)	0.13
Family	45 (13)	14 (8)	31 (17)	0.015
Books	34 (10)	19 (11)	15 (8)	0.34
**Duration (months)**^**1**^**, median (IQR)**	8 (2, 25)	17 (4, 41)	3 (1, 11)	< 0.001
**Reasons ranked 1st for following a LCD**^**2**^				
Weight loss	194 (59)	69 (41)	125 (78)	< 0.001
Better for health	71 (22)	58 (34)	13 (8)	< 0.001
Diabetes management	33 (10)	27 (16)	6 (4)	< 0.001
**Frequency of LCD meal**^**2**^				0.005
Everyday every meal	157 (48)	87 (52)	70 (44)	
Weekdays only	32 (10)	8 (5)^a^	24 (15)^b^	
4–5 days a week	104 (32)	53 (31)	51 (32)	
Up to 3 days a week	18 (6)	7 (4)	11 (7)	
**Portion size during LCD**^**2**^				0.68
Smaller	194 (59)	100 (59)	94 (58)	
No change	64 (19)	35 (21)	29 (18)	
Bigger	72 (22)	34 (20)	38 (24)	
**Weight loss (kg), median (IQR)**^**3**^	8 (4.3–16.5)	9.6 (5.3–19.1)	6.5 (3.6–11.6)	< 0.001
**Weight loss categories (%)**^**3**^				0.001
< 5	82 (29)	35 (22)^a^	47 (39)^b^	
≥ 5 to < 10	80 (28)	47 (29)	33 (27)	
≥ 10 to < 20	74 (26)	41 (26)	33 (27)	
≥ 20 to < 30	33 (12)	25 (16)^a^	8 (7)^b^	
≥ 30	13 (5)	12 (8)^a^	1 (0.8)^b^	
**Support from doctor/GP**^**2**^				< 0.001
Aware and supportive	33 (10)	27 (16)^a^	6 (4)^b^	
Aware but not supportive	11 (3)	9 (5)^a^	2 (1)^b^	
Aware but no indication whether supportive or not	19 (6)	14 (8)^a^	5 (3)^b^	
Not aware that respondent was dieting	267 (81)	119 (70)^a^	148 (92)^b^	
**Support from other HCPs**^**2,4**^				0.74
Aware and supportive	20 (6)	10 (6)	10 (6)	
Aware but not supportive	14 (4)	9 (5)	5 (3)	
Aware but no indication whether supportive or not	5 (2)	2 (1)	3 (2)	
Not aware that respondent was dieting	291 (88)	148 (88)	143 (89)	

Data are n (%) unless otherwise indicated.

^1^Valid sample size, n = 328 (169 current followers, 159 past followers).

^2^Valid sample size, n = 330 (169 current followers, 161 past followers).

^3^Valid sample size, n = 282 (160 current followers, 122 past followers).

^4^Other HCPs included dietitians, nurse, or nutritionist.

^5^Chi-squared test with Bonferroni adjustment for column proportion comparison (categorical data)—different letters represent significant differences between groups.

^6^Mann–Whitney U test for continuous data.

Most current and past LCD followers (n = 200, 71%) reported a weight loss of at least 5% of their baseline body weight, with n = 13 reporting weight loss higher than 30% after LCDs. A minority of respondents (6–10%) reported receiving any support from doctors or other health care professionals (HCP; i.e. dietitian, nurse, nutritionist). Specifically, current followers (n = 27; 16%) had more support from doctors than past followers (n = 6; 4%; Table 4). In contrast, over 80% of both current and past followers reported that their doctors/HCPs did not know that they were following a LCD (Table 4).

**Figure 2:**
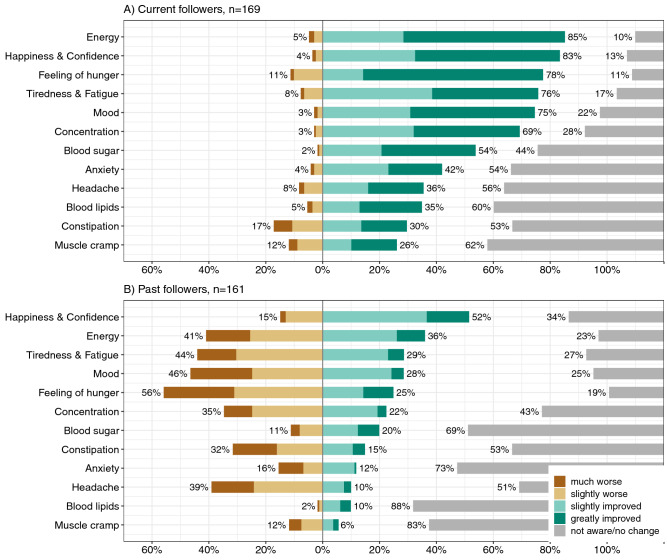
Percentages of current (**A**) and past low-carbohydrate diet followers (**B**) who reported specific experiences during low-carbohydrate diets (n = 330).

Experiences while following a LCD differed between current and past followers (Fig. 2). Among current LCD followers, improvements in all experiences were reported more often than worsening, with an improvement in energy the most common (85%), followed by happiness and confidence (83%; Fig. 2A). A different profile of experiences was reported by past followers, with most of the experiences reported to worsen, with perception of hunger the most often reported negative perceived consequence (56%). Happiness and confidence (52%), blood sugar (20%), and blood lipids (10%) were three experiences reported more often as improving rather than worsening (15%, 11%, and 2% respectively) in past followers (Fig. 2B).

### Psychological constructs and health beliefs of the theory of planned behaviour

The TPB model was applied to explore the attitude, subjective norms, and perceived control over the behaviour. Current followers had the greatest intention to follow a LCD (median 7: strongly agree; IQR 7–7), followed by past followers (median 5: slightly agree; IQR 3–6), whereas non-followers’ intention was in disagreement with following a LCD (median 3: slightly disagree; IQR 1–4; *p* < 0.001; Table 5).

**Table 5:** Theory of planned behaviour constructs (TPB) and their corresponding health beliefs stratified by low-carbohydrate diet status.

Main TPB constructs and their corresponding health beliefs	Possible range^1^	Current followers (n 170)		Past followers (n 184)		Non-followers (n 369)		*P*-value^2^
		Median	IQR	Median	IQR	Median	IQR	
Intention to follow a LCD	1 to 7	7^a^	7, 7	5^b^	3, 6	3^c^	1, 4	< 0.001
Attitude toward following a LCD	1 to 7	7^a^	6, 7	5^b^	4, 6	4^c^	3, 5	< 0.001
Subjective norm	1 to 7	6^a^	4, 7	4^b^	4, 6	4^c^	3, 5	< 0.001
Perceived behavioural control	1 to 7	7^a^	6, 7	6^b^	5, 7	5.5^c^	4, 6	< 0.001
**Behavioural belief composite score**^**3**^	− 84 to 84	36^a^	23, 42	12^b^	0, 27	0^c^	− 13, 10	< 0.001
Good for weight management	− 21 to 21	21^a^	12, 21	10^b^	4, 14	3^c^	0, 10	< 0.001
Risk of micronutrient inadequacy	− 21 to 21	− 3^a^	− 7, − 3	− 9^b^	− 15, − 6	− 12^b^	− 15, − 6	< 0.001
Reduced risk of chronic diseases	− 21 to 21	21^a^	12, 21	12^b^	6, 18	10^c^	4, 15	< 0.001
Side effects e.g. constipation	− 21 to 21	3^a^	0, 10	2^b^	− 4, 10	− 4^c^	− 12, 0	< 0.001
**Normative belief composite score**^**3**^	− 105 to 105	19^a^	8, 32	3.5^b^	− 10, 19	− 11^c^	− 24, 0	< 0.001
Family	− 21 to 21	0^a^	0, 6	0^b^	− 5, 0	− 4^c^	− 10, 0	< 0.001
Friends/colleagues	− 21 to 21	0^a^	0, 8	0^b^	− 2, 6	0^c^	− 4, 2	< 0.001
Doctors	− 21 to 21	0^a^	0, 4	0 ^a^	0, 4	0^b^	− 4, 2	< 0.001
Best-selling books	− 21 to 21	0^a^	− 3, 7	− 2^b^	− 3, 1	− 3^c^	− 4, 0	< 0.001
Internet	− 21 to 21	10^a^	4, 18	4^b^	0, 10	− 2^c^	− 4, 0	< 0.001
**Control belief composite score**^**3**^	− 63 to 63	9^a^	− 6, 22	− 11.5^b^	− 28, 3	− 10^b^	− 26, 0	< 0.001
People around me have carbohydrates in their diets	− 21 to 21	6^a^	− 6, 14	− 7^b^	− 14, 2	− 6^b^	− 12, 0	< 0.001
LCD products expensive and hard to find	− 21 to 21	4^a^	− 2.3, 8.3	0^b^	− 12, 4	0^b^	− 8, 0	< 0.001
No time to cook/prepare meals	− 21 to 21	2^a^	− 2, 3	− 2^b^	− 6, 2	− 2^b^	− 6, 0	< 0.001

*TPB* theory of planned behaviour, *LCD* low-carbohydrate diet.

^1^Possible range 1–7 represents strongly disagree (1) to strongly agree (7); possible range for belief score—positive score of behavioural beliefs means that the respondent is in favour of the behaviour, while negative score means disagreeing with the behaviour. Similarly, positive (or negative) score of normative beliefs means that respondent feels social pressure to execute the behaviour (or not). Positive (or negative) score of control beliefs means that respondent feel (or does not feel) in control of doing the behaviour.

^2^Kruskal–Wallis Test with Bonferroni correction for multiple tests—different letters represent significant differences between groups.

^3^Composite score was the sum of each belief in its category.

#### Attitude and behavioural beliefs (indirect measurement of attitude)

Current followers displayed the most positive attitude toward following a LCD (median 7: strongly agree; IQR 6–7), compared to past followers (5: slightly agree; IQR 4–6) and non-followers (4: neutral; IQR 3–5; *p* < 0.001). Regarding behavioural beliefs, current followers were most in favour with following a LCD (behavioural belief composite score median 36; IQR 23–42), followed by past followers (median 12; IQR 0–27), while non-followers had neutral attitude toward LCD (median 0; IQR − 12.5 to 10; *p* < 0.001; Table 5).

Specifically, behavioural beliefs about “*good for weight management*” and “*reduced risk of chronic disease*” of current followers were at the maximum positive scores (both median 21; IQR 12, 21), representing positive attitude in favour of following a LCD. On the other hand, non-LCD followers had negative attitude toward following a LCD explained by negative scores regarding behavioural beliefs about unfavourable consequences of LCD, namely “*LCD and risk of micronutrient inadequacy*” and “*side effects of LCD*” (Table 5).

#### Subjective norm and normative beliefs (indirect measurement of subjective norm)

Current followers had the greatest subjective norm scores (median 6: agree; IQR 4–7), while past and non-followers showed neutral agreement with subjective norm statements (both median 4) to follow a LCD. Similarly, current followers showed the highest positive normative belief composite scores (median 19; IQR 8–32), followed by past followers (median 3.5; IQR − 10 to 19) reflecting that they perceived positive social pressure to follow a LCD, whereas non-followers had negative composite score reflecting negative social pressure against following a LCD (median − 11; IQR − 21 to 0; *p* < 0.001; Table 5).

Further insight into sources of social pressure (each normative belief, i.e. family, friends, doctors, books, and the internet) showed that the internet was the main positive social pressure (median 10; IQR 4–18) to follow a LCD in current followers and past followers, while the other four normative beliefs showed neutral or negative effect. On the other hand, non-followers perceived negative social pressure from family, books, and the internet against following a LCD (Table 5).

#### Perceived behavioural control and control beliefs (indirect measurement of perceived behavioural control)

Perceived behavioural control is the perception of self-efficacy or ability to carry out a given behaviour. Again, current followers showed the greatest perceived behavioural control over following a LCD (median 7: strongly agree; IQR 6–7) compared to past (median 6: agree; IQR 5–7) and non-followers (median 5.5: between slightly agree to agree; IQR 4–6; *p* < 0.001; Table 5). Regarding control beliefs, current followers felt in control to follow a LCD (control belief composite score median 9; IQR − 6 to 22) while past followers (median − 12; IQR − 28 to 3) and non-followers (median − 10; IQR − 26 to 10; *p* < 0.001) did not (Table 5).

The control belief factor, “*People around me have carbohydrates in their diets*” and “*no time to cook/prepare meals*” were not recognised as barriers by LCD followers (median score 6 and 2 respectively), unlike past and non-followers (median scores ranging from − 2 to − 7; Table 5). The control belief “*LCD products are expensive and less available*” was also not recognised as a barrier by LCD followers, while past and non-followers held a neutral opinion on the matter (Table 5).

### Path analysis explaining low-carbohydrate dieting behaviour

**Figure 3:**
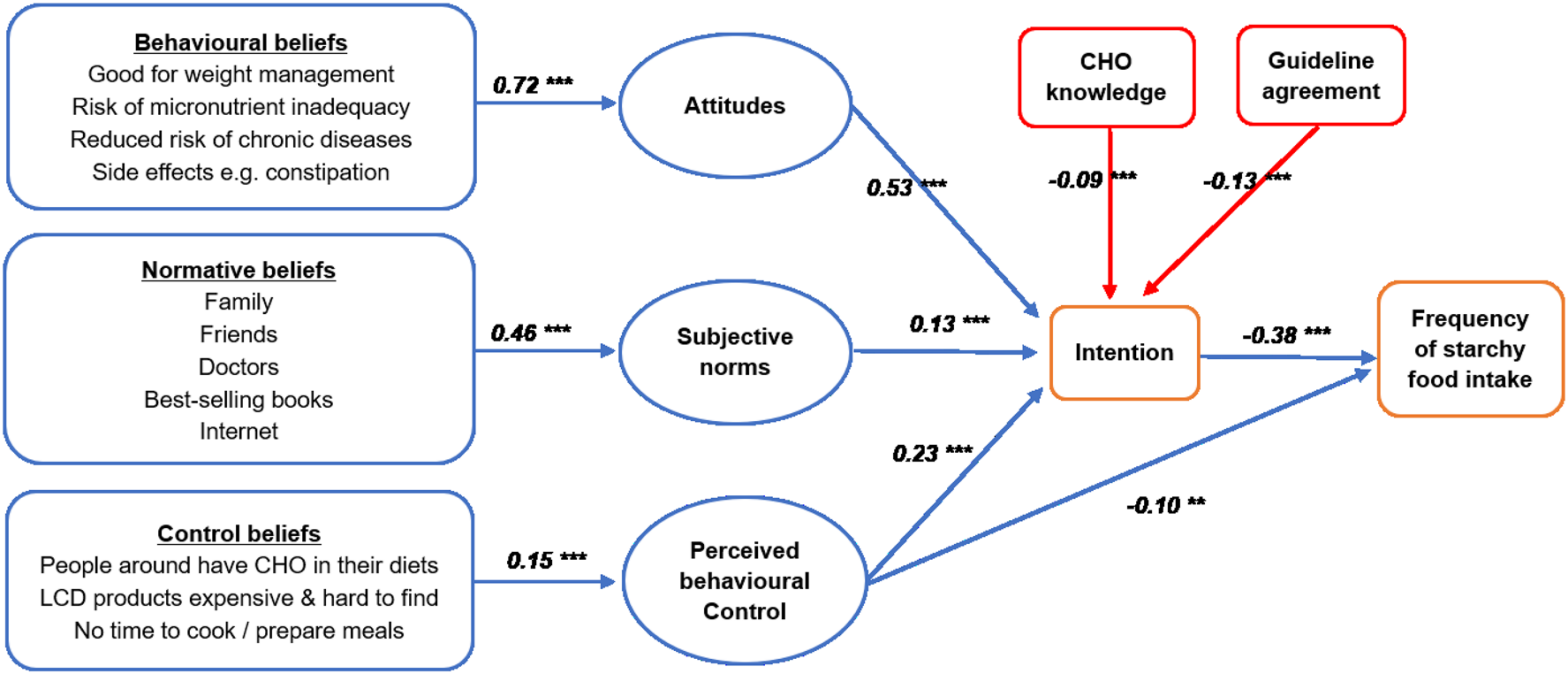
Path analysis of the theory of planned behaviour constructs with their corresponding health beliefs, carbohydrate knowledge and dietary guideline agreement to explain intention and behaviour to follow a low-carbohydrate diet. Values are standardized β-coefficients; **p < 0.01, ***p < 0.001; *LCD* low-carbohydrate diet, *CHO* carbohydrate.

Path analysis was applied to investigate the TBP psychological constructs, carbohydrate knowledge and dietary guideline agreement explaining the intention to follow a LCD (Fig. 3). As proposed in the TPB model, intention is the strongest determinant in performing behaviour, although perceived behavioural control could also influence the behaviour performance. In this study, we defined behaviour using the frequency of starchy food intake as an outcome variable and intention and perceived behaviour control as predictor variables. We found that higher intention to follow a LCD was associated with a lesser frequency of starchy food intake (standardized *β* − 0.38, *p* < 0.001). Perceived behavioural control was also associated with frequency of consumption with lesser effect size (standardized *β* − 0.10, *p* < 0.01; Supplemental Table S1).

The TPB model also indicates that intention can be predicted by attitude, subjective norm, and perceived behavioural control. We found that attitude, subjective norm and perceived behavioural control were also positively associated with intention to follow a LCD (adjusted R^2^ = 0.58, *p* < 0.001), with influence of attitude on intention to follow a LCD the strongest (standardized *β* 0.60, *p* < 0.001), followed by perceived behavioural control (standardized *β* 0.24, *p* < 0.001) and subjective norm (standardized *β* 0.14, *p* < 0.001; Supplemental Table S1, model 1). Adding carbohydrate knowledge and dietary guideline agreement in the model 1, both knowledge score and guideline agreement were negatively associated with intention to follow a LCD (standardized *β* − 0.09 and − 0.13, all *p* < 0.001), with no impact on the coefficients of TPB constructs (Supplemental Table S1, model 2). All health beliefs behind the three TPB constructs showed that behavioural beliefs, normative beliefs, and control beliefs significantly predicted the attitude, subjective norms, and perceived behavioural control respectively (Fig. 3).

## Discussion

To our knowledge, this is the first study that has comprehensively explored the factors behind food choices not only in self-identified LCD followers but also in past and non-LCD followers in UK adults. The application of TPB model demonstrated the hypothesized associations between psychological factors and intention to follow a LCD, which was mostly driven by attitude, aligning with previous meta-analysis of TPB and healthy eating pattern showing that attitude had the strongest association with intention^32^.

Knowledge about carbohydrate foods in terms of nutrition and health, and perceived benefits of dietary guideline could also influence the intention to follow a LCD^22^, and we found that knowledge and guideline agreement had negative influences on intention to follow a LCD. Respondents who had high knowledge about carbohydrates, and their role in nurition and health, were more concerned about the unfavourable consequences of LCDs on micronutrient inadequacy. Foods recommended in LCDs also conflict with dietary guidelines recommendations^25^.

Compared to non-LCD followers, current followers consumed less starchy foods, fruits and vegetables, but more meat, fish, and cheese, similar to a previous study^21^. Current LCD followers consumed starchy foods as little as 0.9 time/day. Assuming that 1 time per day is equivalent to 1 average portion of starchy foods, this would be equivalent to a carbohydrate content of 40 g in 1 cup of cooked pasta or rice, 27 g in 2 slices of breads or in 1 cup of bran cereals, and 54 g in 2 medium size potatoes. This level of carbohydrate intake is to some extent in line with a very low-carbohydrate diet (< 50 g/day)^1, 12^. Although other carbohydrate-rich foods (e.g. cake, ice cream, biscuit, etc.) contribute to overall carbohydrate intake, LCD followers consumed these foods as little as 0.13 times/week (equivalent to never or less than once a month—the minimum frequency of intake in the DTM; this may be partly explained by conscious or unconscious under-reporting of these discretionary items). Cutting out starchy foods and fruits could result in micronutrient inadequacy^23^, especially if this is not guided appropriately^35^. Although supplementation could top up vitamins and minerals intake during LCD, only 10% of LCD followers reported use of multivitamins/minerals supplements.

Our findings also highlighted that there were differences in perceived experiences during LCD between current and past followers. The experience of current followers highlighted improvement in all experiences more often than worsening, often (but not always) unlike past followers. While some of these contrasting experiences may explain why some people do well and can maintain specific diet, it also provides confirmation that there is not one single best diet for all. Weight loss, health, and diabetes management were the most reported motivations to follow a LCD in our study, similar to previous studies^8, 34^. These motivations could be influenced by perceived benefits of LCDs for weight loss and prevention for chronic diseases. Diets restricting carbohydrate intake take a wide range of format, and can work for some people, as shown by the diverse and opposite perception of symptoms that improved or worsened during dieting. More importantly, either for weight loss or because of pre-existing health risk, over 80% of LCD followers reported that their doctor/HCP did not know that they were following a LCD, higher than previously reported in a study from the USA (50%)^24^ highlighting a potential concern regarding engagement with primary care and allied health professionals regarding diet and lifestyle. The internet was the most frequently reported source of information and inspiration regarding LCDs, which in part could be explained by the availability of successful weight loss stories online^5, 6^.

Our findings could help inform practice as we highlighted several challenges associated with following a LCD. The access to support from doctors or dietitians was low, and the internet was the most commonly cited source guiding or influencing LCD behaviour, indicating that there is limited reliable resources for those who want to follow a LCD. Provision of reliable resources online or increased access to healthcare support on how to follow a LCD, especially in terms of nutrient-dense food selection, would support knowledge and ability to follow a LCD while preventing the risk of inadequate vitamin and mineral intake^23, 35, 36^. Although our study found that individuals practicing medicine (doctors) were more likely to follow a LCD, this was not true for dietitians, food scientists, or other healthcare professionals. The motivations for following a LCD, in individuals practicing medicine (e.g. knowledge, income), is an interesting area to be explored further. Determinants for dietary behaviours or food choices have mostly been debated in Western countries, however, this is a debate also relevant to other regions (e.g. Asia) where different barriers and opportunities could arise depending on local food systems and culture. This is something that remains to be investigated.

There are limitations in this study. The aims of the study were exploratory rather than to determine prevalence. As the nature of the online survey, representativeness cannot be completely assured: the proportion of LCD followers could not be inferred as representing the prevalence of LCD use in the UK or elsewhere. The dietary assessment was limited to frequency of consumption, it was unable to generate the amount of carbohydrate intake. Therefore, we could not explore the number of LCD followers who were in line with the more conservative LCD definition (e.g. carbohydrate of less than 26% of energy intake per day)^1, 12^. The lack of a standard definition for LCDs may impact on the study findings. In practice, reducing carbohydrate intake may imply (or not) caloric restriction; to balance for a lower contribution of carbohydrate towards energy intake, either fat or protein may be increased. The perceptions (health beliefs, lived experience) of these very different variations of a LCD, from an energy, macronutrient and possibly micronutrient point of view would be expected to differ and deserves further attention. The TPB has been used to predict intention and future behaviour^31, 32^, for example, the intention to eat a healthful diet and the actual behaviour of eating a healthful diet further down in time. Due to the cross-sectional design of this study, we cannot reflect on actual behaviour change post survey.

## Conclusions

The current study provides better understanding of the nutritional behaviour and motivations to follow a LCD, with losing weight and health listed as the primary reasons. It also provides an overview of the experience of dieters, highlighting both positive and negative experiences while following the diet. The findings could help design individually preferred weight management programmes, moving away from one-size fits all. Application of the TPB helps elucidate factors explaining the intention to follow a LCD among UK adults. Positive attitude (e.g. perception that a LCD is effective for weight management and reduced risk of chronic disease) is the most powerful determinant for intention to follow a LCD, while carbohydrate knowledge and agreeing with dietary guideline are negative determinants. It is of concern that a majority of LCD followers do not have explicit support from HCPs and that a minority takes multivitamins/minerals supplement despite the restrictive nature of the diet. As there is no specific best weight loss diet, LCDs may suit some people’s preference^2, 3, 37^, indicating a place for better professional guidance and support on weight loss diet, and improved knowledge about high nutrient-dense food selection to minimize the health risk generated from LCDs. Weight regain after a LCD as well as reasons why past followers stopped, or why non-followers chose not to follow the diet deserve further investigation.

## Methods

### Study design and population

A cross-sectional online survey was conducted between February to December 2019 in a sample of UK adults, aged 18 years and older. The survey included participants who self-identified either as LCD followers, or non-followers. Entering the survey was incentivized with a £50 Amazon voucher prize, drawn every 100 entries. Participants were recruited via social media i.e. Twitter, Facebook, Instagram, and web service for online survey recruitment (i.e. callforparticipants.com and prolific.co). Written informed consent was obtained from all the participants. The study was approved by the University of Glasgow Research Ethics Committee (project no. 200180040).

### Sample size

The sample size was calculated using the equation for estimated proportion for an infinite population^38^. With 95% confidence level (alpha 0.05), 2.5% absolute precision (margin of error), and a design effect of one as suggested by Gorstein et al., a margin of error should not be more than 3 percentage points if an estimated prevalence is less than 20%^39^. We assumed an overall 10% prevalence of LCD followers in the UK, based on a media poll in the UK^7^ showing that 7% of men and 10% of women had tried this diet. Based on this calculation, the minimum sample required was 555 UK adults, which should provide 56 respondents as LCD followers. We aimed to recruit over this number to ensure a sufficient sample size, assuming 20% of responses would be incomplete. Therefore, a final number of at least 694 UK adults was targeted, expecting at least 70 LCD followers.

### Questionnaire instrument

All data collected in this study were self-reported data. The questionnaire gathered socio-demographics data (i.e. age, sex, weight and height, education, income, ethnicity, smoking status, co-morbid diseases, supplement use). BMI was calculated by weight (kg) divided by the square of height (m). Other components included self-reported “LCD status” (current, past and non-follower), dietary intake, knowledge and perceived understanding of carbohydrates, and awareness of current national dietary guidelines. The questionnaire also included the psychological constructs determining behavioural intention to follow a LCD, with the application of the TPB model (Supplementary File).

#### Dietary assessment

Dietary intake was assessed using the Dietary Targets Monitor (DTM)^40^. The DTM is an easily administered, short food frequency list, which was designed to assess habitual intake of the foods in comparison to Dietary Targets for Scotland^40^. The commonly consumed foods listed in the DTM were fruits and vegetables, carbohydrate-rich foods (e.g. breads, cereals, pasta, potatoes), meat, chicken, fish, pastries and savoury snacks. The DTM can estimate the amount of food and nutrient intake for fruits and vegetables (g/day), fish (g/week), and fat and saturated fat (g/day) using calibrated formulas^40^. The remaining individual food items can be reported as frequency of consumption (times/day or times/week). Further details of the DTM are described in Lean et al.^40^.

#### Nutritional behaviour of low-carbohydrate diet followers

Nutritional behaviour of current and past LCD followers was collected as following: names of LCD followed, source of information about LCD, reasons to follow a LCD, timescale of following a LCD, meal portion size while following a LCD, frequency of eating LCD (e.g. every day, only weekday, only weekend, etc.), experience of benefits and adverse events during LCD, as well as source of support while following a LCD (e.g. healthcare professionals, including doctors and dietitians).

#### Perceived understanding and knowledge about carbohydrates

Perceived understanding about carbohydrate was assessed using the question “Do you understand what the term ‘carbohydrate’ means?” with four response answers: (1) yes, very good idea; (2) yes, vague idea; (3) no, not really, but I know what foods contain carbohydrate; and (4) no, not at all. Knowledge about carbohydrate-rich food was derived from 11 items designed around type and source of carbohydrate, food processing, and nutrition. Each item was scored 1 point for correct answer, − 1 for incorrect, and zero point if participants selected ‘do not know’. The score was summed, giving a range from − 11 to 11.

#### Dietary guideline awareness and agreement

To assess awareness of the current dietary guidelines, participants were asked whether they had heard of the UK Eatwell plate/guide (or MyPlate, USA) and whether they were following one of these guidelines. They were also asked whether they agreed with these guidelines, including agreement on each of the four statements in the UK Eatwell guide (i.e. carbohydrate foods, fruits and vegetables, oils, and foods high in fat, salt and sugar). To assess the level of agreement on UK Eatwell guide, each of four statements scored 1 point if respondents answered ‘agree’, − 1 for ‘disagree’, and no point for ‘not sure’. The score was summed, giving a range from − 4 to 4.

#### The theory of planned behaviour

The TBP model was applied to assess the low-carbohydrate dieting behaviour and the intention to follow a LCD. The model includes *intention* to follow a LCD, *attitude* towards following a LCD, *subjective norm*, and *perceived behavioural control* over low-carbohydrate dieting behaviour. In addition, there are beliefs underlying attitude, subjective norm, and perceived behavioural control^41^.

1. *Behavioural beliefs* are the beliefs about the expected outcomes (e.g. good or bad) of the behaviour and then produce a favourable or unfavourable attitude towards the behaviour. Four behavioural beliefs were investigated as follow: *LCD for weight management, risk of micronutrient inadequacy following a LCD, reduced risk of chronic diseases following a LCD, and the side-effects of LCD.* There was also an *outcome evaluation* corresponding to each behavioural belief. Outcome evaluation indicates respondents’ expression either positive or negative evaluation of the behavioural belief. For example, a behavioural belief of *‘following a LCD can keep body weight in a healthy range’* corresponds to an outcome evaluation of *‘having a normal body weight is desirable or undesirable’*.
2. *Normative beliefs* are the beliefs about the expectation or influence of others (normative referent) on performing the behaviour and result in perceived social pressure or subjective norm. Five normative referents were assessed as follow: *family, friends and colleagues, doctor, books, and the internet*. There was also a *motivation to comply* corresponding to each normative belief. Motivation to comply indicates the importance of normative referent on doing a behaviour. For example, a normative belief of *‘family think that I should follow a LCD’* corresponds to a motivation to comply of *‘what family think I should do matters to me’*.
3. *Control beliefs* are the beliefs about the factors that could facilitate or inhibit the behaviour and result in perceived behavioural control. Three control beliefs were assessed as follow: *joining mealtime with other people who have starchy foods in their diets, high price and lack of ready to eat LCD foods, and a lack of time to cook or prepare meals.* There was also a *power of control factor* corresponding to each control beliefs. Power of control factor indicates the power of control belief to influence the behaviour. For example, a control belief of ‘*people round me have starchy foods in their diets*’ corresponds to a power of control factor of *‘joining mealtime with other people who have starchy foods makes it more difficult to follow a LCD’.*

Belief statements were derived from review of the literature as well as online discussion forum and social media (Twitter, Facebook groups). The list of belief statements was then given to a small convenient sample to check and complete. The final TPB questionnaire consisted 29 question-items, assessing intention, attitude, subjective norm, perceived behavioural control, and the three types of beliefs above. The strength of intention, attitude, subjective norm, perceive behavioural control, and all beliefs were measured using 7-point bipolar rating scales, ranging from strongly disagree (1) to strongly agree (7). The statements about outcome evaluation, motivation to comply, and power of control factor were measured using the same responses but different scoring system, ranging from − 3 for strongly disagree to + 3 for strongly agree.

Before analysis, each behavioural belief was multiplied by its corresponding outcome evaluation, normative beliefs multiplied by motivation to comply, and control beliefs multiplied by power of control factor, as described in Francis et al*.*^42^. Then each belief in its category was summed to create a composite score, for example four behavioural beliefs were summed to create a *behavioural belief composite score*. Similar process was done for *normative belief composite score* and *control belief composite score*. Positive score of behavioural beliefs means that the respondent is in favour of the behaviour, while negative score means disagreeing with the behaviour. Similarly, positive (or negative) score of normative beliefs means that respondent feels social pressure to execute the behaviour (or not). Positive (or negative) score of control beliefs means that respondent feel (or does not feel) in control of doing the behaviour.

#### Questionnaire validation

The questionnaire was assessed for face and content validity among colleagues in Human Nutrition, University of Glasgow (n = 18), who were informed about the objectives of the survey. Feedback focused on readability, feasibility, clarity of wording and layout and style, and whether it was an objective tool to answer the questions. An average time spent on questionnaire was 22 min (SD 3). The final questionnaire version was created using the Online Survey System (www.onlinesurveys.ac.uk). The online version of the questionnaire was tested in a group of people outside nutrition field (n = 5) for readability and understanding before launching.

### Statistical analysis

Descriptive statistics were used to describe sample characteristics, knowledge score, awareness of dietary guidelines, dietary intake and TPB constructs. Group differences between LCD followers and non-LCD followers were assessed with *t*-test or ANOVA for continuous data and Chi-square test for categorical data. Non-parametric statistics were used where appropriate.

Path analysis^43^ was used to test the hypothesized TPB model, using multiple linear regression analysis to determine psychological constructs of the TPB (independent variables: attitude, subjective norm, and perceived behavioural control) that influence participants’ intention (dependent variable) towards following a LCD (model 1, the independent variables were adjusted to each other). Then in the model 2, carbohydrate knowledge and guideline agreement were added in the model 1 (further adjustment), to determine their associations with intention to follow a LCD, and to investigate whether these variables affect the associations between TPB constructs and the intention.

Path analysis was also applied for analysis of the influence of intention (independent variable) on low-carbohydrate dieting behaviour (dependent variable). We defined behaviour as the frequency of consumption of starchy foods and used it as a dependent variable in a linear regression analysis. Perceived behavioural control variable was also added in the model predicting behaviour as shown in the TPB.

### Ethical declaration

Written informed consent was obtained from all the participants prior to their inclusion in the study. The study was approved by the University of Glasgow Research Ethics Committee (project no. 200180040), and have been performed in accordance with the ethical standards laid down in the 1964 Declaration of Helsinki.

## Supplementary information

Supplementary file 1
