# Supplementary material for: Carbohydrate knowledge, dietary guideline awareness, motivations and beliefs underlying low-carbohydrate dietary behaviours
Source: Sci Rep. 2020 Sep 2;10:14423. doi: 10.1038/s41598-020-70905-2 (PMC7468104; doi:10.1038/s41598-020-70905-2)
Supplement: Supplementary file 1 [file 41598_2020_70905_MOESM1_ESM.pdf]

Supplementary Information

Carbohydrate knowledge, dietary guideline awareness, motivations and beliefs underlying low-carbohydrate dietary behaviours

Chaitong Churuangsuk [1] - https://orcid.org/0000-0003-4373-6395

Michael E.J. Lean [1] - https://orcid.org/0000-0003-2216-0083

Emilie Combet [1 *] - https://orcid.org/0000-0002-9302-8971

1. Human Nutrition, School of Medicine, Dentistry, and Nursing, College of Medical, Veterinary and Life Sciences, University of Glasgow, Glasgow, UK

*** Corresponding Author**

Dr Emilie Combet

Room 2.22, Level 2, New Lister Building

10-16 Alexandra Parade, Glasgow Royal Infirmary, Glasgow, UK, G31 2ER.

Email: emilie.combetaspray@glasgow.ac.uk

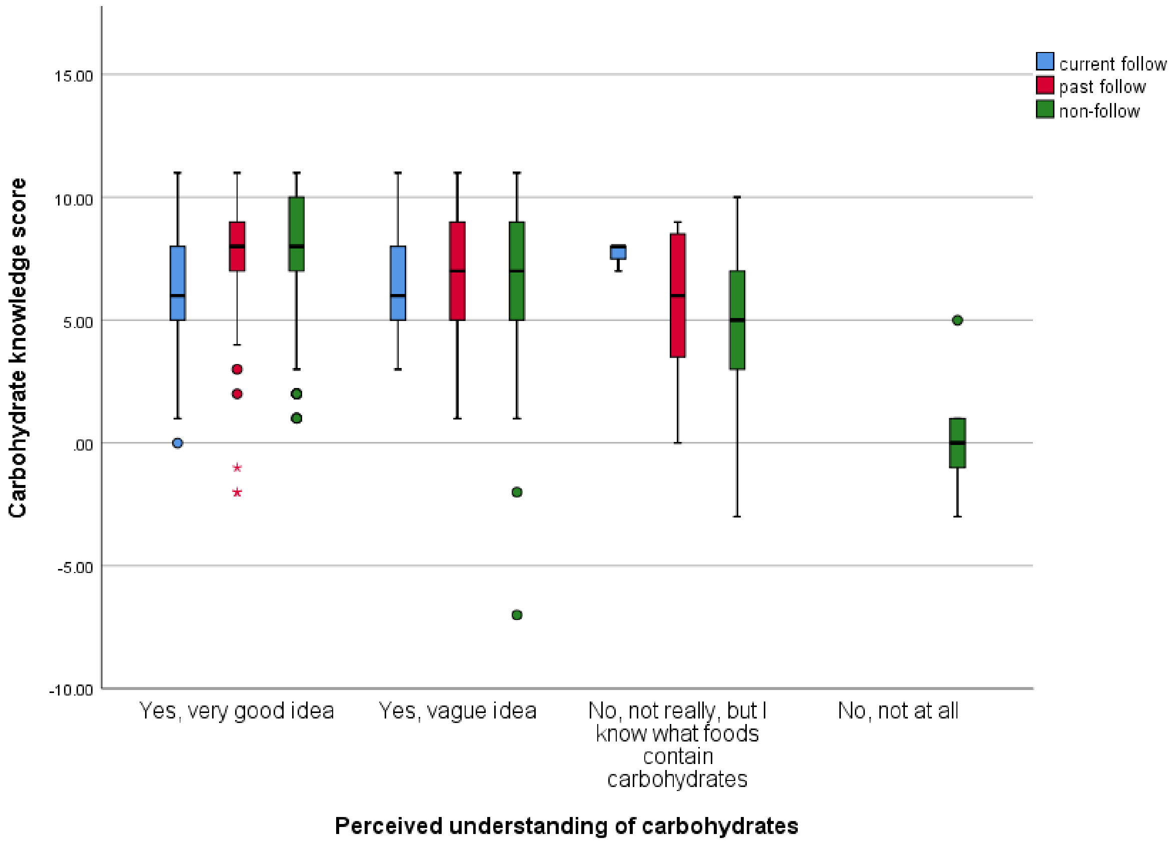

Supplemental Figure S1. Boxplot of carbohydrate knowledge score by level of perceived understanding of carbohydrates stratified by low-carbohydrate diet status.

Supplemental Table S1. Theory of planned behaviour constructs (attitude, subjective norm, perceived behavioural control), carbohydrate knowledge, and dietary guideline agreement influencing intention to follow a low-carbohydrate diet, and the intention and perceived behavioural control predicting the LCD behaviour.

Dependent variables (outcomes)	Independent variables (predictors)	Standardized β coefficient	t	p-value	
Behaviour: frequency of starchy food consumption	Intention	-0.38	-10.2	*<0.001*	adjusted R^2 0.18, ANOVA p<0.001
	Perceived behavioural control	-0.10	-2.7	*0.008*	
Intention (model 1)	Attitude	0.60	21.5	*<0.001*	adjusted R^2 0.58, ANOVA p<0.001
	Subjective norms	0.14	5.3	*<0.001*	
	Perceived behavioural control	0.24	9.5	*<0.001*	
Intention (model 2)	Attitude	0.53	18.3	*<0.001*	adjusted R^2 0.60, ANOVA p<0.001
	Subjective norms	0.13	4.9	*<0.001*	
	Perceived behavioural control	0.23	9.1	*<0.001*	
	Carbohydrate knowledge	-0.09	-3.6	*<0.001*	
	Guideline agreement	-0.13	-4.7	*<0.001*	
Attitude	Behavioural belief composite score	0.72		*<0.001*	
Subjective norm	Normative belief composite score	0.46		*<0.001*	
Perceived behavioural control	Control belief composite score	0.15		*<0.001*	

Section 1 - Carbohydrates and Guidelines

4. **Do you understand what the term ‘carbohydrate’ means?** ✱ *Required*

- ○ Yes, very good idea
- ○ Yes, vague idea
- ○ No, not really, but I know what foods contain carbohydrates
- ○ No, not at all

5. **Please provide UP TO 3 words or groups of words that come to mind when thinking about ‘carbohydrates’ in relation to nutrition & health:**

1.	
2.	
3.	

6. **Please provide UP TO 3 words or groups of words that come to mind when thinking about ‘low-carbohydrate diets’ in relation to nutrition & health:**

1.	
2.	
3.	

7. **Please provide UP TO 3 words or groups of words that come to mind when thinking about ‘high-carbohydrate diets’ in relation to nutrition & health:**

1.	
2.	
3.	

Section 1 - Carbohydrates and Guidelines (cont)

These are examples of dietary guidelines/recommendations

- Eatwell plate or Eatwell guide (UK)
- MyPlate (USA)

8. **Have you heard about any of the above dietary guidelines/recommendations?** ✱ *Required*

- ○ No, I have NOT heard about any of the above before.
- ○ Yes, I have heard of these before

8.a. If **YES**, which one? *Tick all that apply*

- ☐ Eatwell plate or Eatwell guide (UK)
- ☐ MyPlate (USA)
- ☐ Other
- ☐ Not sure

8.a.i. If you selected Other, please specify:

____

8.b. If **YES.** Are you following any of the guideline(s) above?

- ○ Yes
- ○ No

8.b.i. If YES, Which one?

- ☐ Eatwell plate or Eatwell guide (UK)
- ☐ MyPlate (USA)
- ☐ Other dietary guideline

8.b.i.a. If you selected Other, please specify:

____

8.c. Do you agree with their recommendations? *Tick ONE*

- ○ Strongly agree
- ○ Slightly agree
- ○ Neutral
- ○ Slightly disagree
- ○ Strongly disagree
- ○ I am not sure.

8.c.i. Why do you agree or disagree? *Optional*

____

9. **The following recommendations are outlined in the UK Eatwell Guide. What do you think about the following statement?** *Tick ONE for each statement.*

	✱ *Required*		
	Agree	Disagree	I'm not sure
Base meals on potatoes, bread, rice, pasta or other starchy carbohydrates. Choose wholegrain where possible.	○	○	○
Eat at least five portions of a variety of fruit and vegetables a day.	○	○	○
Choose unsaturated oils and spreads and eat in small amounts such as vegetable, rapeseed, olive and sunflower oils.	○	○	○
Eat foods high in fat, salt and sugar less often and in small amounts. These foods include chocolate, cakes, biscuits, sugary soft drinks, butter, ghee and ice cream	○	○	○

9.a. *** If you disagree with any of the statements above -> why?**

Carbohydrates

10. **What do you think regarding the following statements?** *Tick ONE for each statement.*

	✱ *Required*		
	True	False	I'm not sure
Carbohydrates are a food group which comprises sugars, starches and fibres found in grains, vegetables, fruits and milk products.	○	○	○
Carbohydrates are NOT the body's main source of energy.	○	○	○
Carbohydrates include any sugars, including those coming from sweets, candy, syrup, honey and fruit juice.	○	○	○
Added sugars are sugars that are added to foods and drinks. They include sugars such as glucose, fructose, sucrose or table sugar, syrup, as well as sugars naturally present in honey, fruit juices and fruit juice concentrates.	○	○	○
Starch (from potatoes, bread, rice, pasta) is NOT a carbohydrate.	○	○	○
Fibre (dietary fibre) is found mostly in wholegrain as well as vegetables, pulses and fruits.	○	○	○
Whole grain starchy foods (e.g. wholegrain bread, breakfast cereals, brown rice and whole-wheat pasta) are a good source of fibre, B-vitamins and minerals.	○	○	○
Refined grains have been milled, a process that removes the bran and germ. This PROCESS can remove dietary fibre, minerals, and many B vitamins.	○	○	○
White breads and pasta (made from white flour), and polished white rice are refined starchy foods (refined carbohydrates).	○	○	○
Gluten is a protein found in wheat and some people are allergic to gluten.	○	○	○
High consumptions of whole grains and fibre may increase the risk of obesity, heart disease, diabetes and some cancer types.	○	○	○

Low-carbohydrate diets

11. **Have you heard of a low-carbohydrate diet?** *Tick ONE* ✱ *Required*

○ Yes
○ No, I have not heard nor am I currently following this diet.

11.a. If **YES. Have you tried a low-carbohydrate diet before?** *Tick one*

○ Yes, I am following a low-carbohydrate diet at the moment
○ Yes, I have heard and tried this diet before, but I am not following it at the moment.
○ I have heard about this diet but never tried it.

11.b. If **YES, what low-carbohydrate diets have you heard of or are you following?** *Tick all that apply*

	Now following	Followed in the past	Heard of	Never heard
1. Atkins Diet	○	○	○	○
2. Zone diet	○	○	○	○
3. Sugar Busters diet	○	○	○	○
4. South Beach Diet	○	○	○	○
5. Harcombe Diet	○	○	○	○
6. Paleolithic diet (Paleo diet)	○	○	○	○
7. Ketogenic Diet (Keto diet)	○	○	○	○
8. Gluten-free diet	○	○	○	○
9. a gluten-free diet plus any low-carbohydrate diets	○	○	○	○
10. no specific named diet/my own variation of low-carbohydrate diet	○	○	○	○
11. Do not know the name	○	○	○	○
12. Other kind (please specify below)	○	○	○	○

11.b.i. if you select 'other kind' please specify in the box below

11.c. If **YES**, **who are your main sources of advice/inspiration regarding your diet.**
Rank TOP 3 sources by numerical order 1 to 3. (1 being your biggest influence, 3 being your lowest)

Please don't select more than 1 answer(s) per row.

Please select exactly 3 answer(s).

Please don't select more than 1 answer(s) in any single column.

	1	2	3
Family members	☐	☐	☐
Friends/Peers/Colleagues	☐	☐	☐
Books/Magazines	☐	☐	☐
Internet website (Blogs, online support web forums)	☐	☐	☐
Media (TV, radio, press)	☐	☐	☐
Social medias (facebook, twitter, Pinterest, Instagram, etc)	☐	☐	☐
Health care professionals (GP/doctor, nurse, dietitian, etc)	☐	☐	☐
Organisations (WHO, NHS, government, associations)	☐	☐	☐
Other(s)	☐	☐	☐

11.c.i. if you select 'other' please specify in the box below

12. **What would you reduce in your diet, if you decided to follow a low-carbohydrate diet?** *Tick all that apply to you* ✱ *Required*

- ☐ Fried potato products (e.g. chips, crisps)
- ☐ All potato product/food including mashed/steamed/baked/fried potatoes or crisps
- ☐ all bread
- ☐ refined bread (e.g. white bread)
- ☐ all rice
- ☐ polished rice (e.g. white rice)
- ☐ all pasta
- ☐ refined pasta
- ☐ breakfast cereals / porridge
- ☐ gluten-containing starchy foods such as whole-wheat breads.
- ☐ bakery products (cakes, scones, or pastries, etc) and snacks (biscuits, crisps)
- ☐ items with added sugars including soft/fizzy drinks, sweets, chocolates, ice-cream.
- ☐ fruits and fruit juices
- ☐ alcoholic drinks including beer
- ☐ Other

12.a. If you selected Other, please specify:

If you are either following or have followed a low-carbohydrate diet in the past, such as the Atkins Diet or any of the diets stated in question 11b.

Click 'YES'

If not, click 'NO'

13. **Are you following / have you followed a low-carbohydrate diet in the past?** ✱ *Required*

○ Yes
○ No

Low-carbohydrate diets practice

14. **Please rank the TOP 3 reasons, from 1 to 3, for opting to follow a low-carbohydrate diet (1 is the most important reason, 3 the least).** ✱ *Required*

Please don't select more than 1 answer(s) per row.

Please select exactly 3 answer(s).

Please don't select more than 1 answer(s) in any single column.

	1	2	3
For losing weight	☐	☐	☐
For weight maintenance (if you have lost weight using other approaches)	☐	☐	☐
For diabetes management	☐	☐	☐
I am allergic to gluten or wheat products	☐	☐	☐
Because it is better for my health	☐	☐	☐
no particular reason	☐	☐	☐
Other (please specify)	☐	☐	☐

14.a. If you selected Other, please specify:

15. How long have you followed a low-carbohydrate diet until now (or from start to end of your dieting)?

	✱ *Required*
the date you have started:	(dd/mm/yyyy)
the date you have stopped: (If still following the diet, enter today date)	(dd/mm/yyyy)

16. **How consistently (on average) do/did you follow a low carbohydrate diet?** ✱ *Required*

- ○ Everyday, every meals
- ○ weekdays only
- ○ weekends only
- ○ 4/5 days a week, depending on where I am / who I am with
- ○ up to 3 days a week, depending on where I am / who I am with
- ○ Other

16.a. If you selected Other, please specify:

17. What is / has been your experience of following a low carbohydrate diet?

	✱ Required					
	Not aware/I don't know	Much worse	Slightly worse	No change	Slightly improved	Greatly improved
1. Feelings of hunger	○	○	○	○	○	○
2. Energy	○	○	○	○	○	○
3. Mood	○	○	○	○	○	○
4. Concentration	○	○	○	○	○	○
5. Headache	○	○	○	○	○	○
6. Anxiety	○	○	○	○	○	○
7. Tiredness, fatigue	○	○	○	○	○	○
8. Muscle cramps	○	○	○	○	○	○
9. Constipation	○	○	○	○	○	○
10. Blood sugar	○	○	○	○	○	○
11. Blood lipids (e.g. total cholesterol, LDL, HDL, triglyceride)	○	○	○	○	○	○
12. Happiness and confidence	○	○	○	○	○	○

17.a. Other (please specify)

18. What was your weight before starting the low-carbohydrate diet?

	✱ Required	
your weight before starting the low-carbohydrate diet		Kilograms

19. How much weight you have lost since you began following a low-carbohydrate diet (or from start to end of your dieting)?

	✱ Required	
amount of weight loss		Kilograms

20. What is your current weight?

	✱ Required	
your current body weight		Kilograms

Low-carbohydrate diets practice (cont)

21. **While following a low-carbohydrate diet, how is / was the portion size or total volume of most of your meals, compared to what you ate before your low-carbohydrate diet?** *Tick ONE* ✱ *Required*

- ○ Much smaller
- ○ Slightly smaller
- ○ No change
- ○ Slightly bigger
- ○ Much bigger

22. **Is (Was) your GP aware that you are following (or have followed in the past) a low carbohydrate diet?** ✱ *Required*

- ○ Yes
- ○ No
- ○ Not sure

22.a. If **YES**, is (was) he/she supportive of following a low-carbohydrate diet?

- ○ Yes, he/she is supportive.
- ○ No, he/she is not.
- ○ He/she did not comment.

22.b. Has your GP made any recommendation to you regarding your diet?

- ○ No.
- ○ Yes, please specify in a space below.

22.b.i. If you selected Yes, please specify:

23. **Have you seen a dietitian/nurse/nutritionist regarding your diet?** ✱ *Required*

- ○ Yes
- ○ No
- ○ Not sure

23.a. If Yes, who did you speak to?

- ☐ Dietitian
- ☐ Nurse
- ☐ Nutritionist
- ☐ Other

23.a.i. If you selected Other, please specify:

23.b. If **YES**, is (was) he/she supportive of following a low-carbohydrate diet?

- ○ Yes, he/she is supportive.
- ○ No, he/she is not.
- ○ He/she did not comment.

23.c. Has he/she made any recommendation to you regarding your diet?

- ○ No.
- ○ Yes, please specify in a space below.

23.c.i. If you selected Yes, please specify:

24. **Please use this space to add any comments on your low-carbohydrate diet plan.** *Optional*

Section 2 - Your DIET

25. **Click *'view as separate questions instead'* above,** if you are using mobile phone. **How often do you eat these foods.** *Tick* **one** *column for each food (per day or per week or per month).*

	✱ *Required*								
	6+ times/day	4-5 times/day	2-3 times/day	Once/day	5-6 times/week	2-4 times/week	Once/week	1-3 times/month	<1 or never/month
Breakfast cereal, porridge	○	○	○	○	○	○	○	○	○
slices of bread/rolls	○	○	○	○	○	○	○	○	○
Fresh fruit	○	○	○	○	○	○	○	○	○
Cooked green vegetables (fresh or frozen)	○	○	○	○	○	○	○	○	○
Cooked root vegetables such as carrots (fresh or frozen)	○	○	○	○	○	○	○	○	○
Raw vegetables or salad (including tomatoes)	○	○	○	○	○	○	○	○	○

26. **How often do you eat these foods.** *Tick* ***one*** *column for each food (per day or per week or per month). (Continued)*

	✱ *Required*								
	6+ times/day	4-5 times/day	2-3 times/day	Once/day	5-6 times/week	2-4 times/week	Once/week	1-3 times/month	<1 or never/month
Chips	○	○	○	○	○	○	○	○	○
Potatoes (other than chips/crisps)	○	○	○	○	○	○	○	○	○
Pasta or rice	○	○	○	○	○	○	○	○	○
Meat (i.e. beef, pork, lamb)	○	○	○	○	○	○	○	○	○
Processed meats (i.e. sausages, ham)	○	○	○	○	○	○	○	○	○
Chicken	○	○	○	○	○	○	○	○	○

27. **How often do you eat these foods.** *Tick **one** column for each food (per day or per week or per month). (Continued)*

	✱ *Required*								
	6+ times/day	4-5 times/day	2-3 times/day	Once/day	5-6 times/week	2-4 times/week	Once/week	1-3 times/month	<1 or never/month
White fish (i.e. seabass, cod)	○	○	○	○	○	○	○	○	○
Oil rich fish (i.e. salmon, Mackerel)	○	○	○	○	○	○	○	○	○
Cheese	○	○	○	○	○	○	○	○	○
Beans or pulses (i.e. peanut, almonds, etc)	○	○	○	○	○	○	○	○	○
Sweets, chocolates	○	○	○	○	○	○	○	○	○
Ice cream	○	○	○	○	○	○	○	○	○

28. **How often do you eat these foods.** *Tick **one** column for each food (per day or per week or per month). (Continued)*

	✱ *Required*								
	6+ times/day	4-5 times/day	2-3 times/day	Once/day	5-6 times/week	2-4 times/week	Once/week	1-3 times/month	<1 or never/month
Crisps, savoury snacks	○	○	○	○	○	○	○	○	○
Fruit juice (NOT squash)	○	○	○	○	○	○	○	○	○
Soft/fizzy drinks (i.e. Irn-bru, coke)	○	○	○	○	○	○	○	○	○
Sugar reduced/ sugar free soft drinks (i.e. sugar free Irn-bru, diet coke)	○	○	○	○	○	○	○	○	○
Cakes, scones, sweet pies or pastries	○	○	○	○	○	○	○	○	○
Biscuits	○	○	○	○	○	○	○	○	○

Your DIET

29. **What kind of bread do you usually eat?** *Tick ONE*

- ○ white
- ○ brown, granary, wheat meal
- ○ wholemeal
- ○ no usual type
- ○ do not know
- ○ do not eat bread at all
- ○ Other kind (please specify)

29.a. If you selected Other, please specify:

30. **What do you usually spread on bread / toast / crackers?** *Tick ONE*

- ○ butter
- ○ hard/block margarine
- ○ soft margarine
- ○ reduced fat spread
- ○ low fat spread
- ○ Nutella or chocolate spreads
- ○ no usual type
- ○ do not know
- ○ Nothing
- ○ Other

30.a. If you selected Other, please specify:

31. **What kind of milk do you usually use?** *Tick ONE*

- ○ whole milk
- ○ semi-skimmed
- ○ skimmed
- ○ dairy alternatives (e.g. soya, rice)
- ○ no usual type
- ○ do not know
- ○ do not drink milk at all
- ○ plant milk / milk alternative
- ○ Other kind (please specify)

31.a. If you selected Other, please specify:

32. **Do you usually take sugar in:**

	Yes	No	I do not drink it	How much? (e.g. 1 teaspoon/sachet)
Tea	○	○	○	
Coffee	○	○	○	

33. **Which type of breakfast cereal do you normally eat?**

- ○ high fibre (e.g. All Bran, Branflakes, Shredded Wheat, Muesli, Porridge, Weetabix)
- ○ other cereals (e.g. Cornflakes, Rice Krispies, Special K, Sugar Puffs, Honey Snacks)
- ○ no usual type
- ○ do not eat breakfast cereals at all

Attitude towards low-carbohydrate diets

This section will ask your opinion about low-carbohydrate diets.

- Please note that, **low-carbohydrate** diets in this section refer to eating **LESS STARCHY FOODS** (bread, pasta, rice, cereals) and **SWEET FOODS** (e.g. biscuits, cakes, sweets).
- Please tick the level of agreement which apply to your answers OR tick if you do not know.
- This questionnaire will use the term 'Low Carb Diet' instead of 'low-carbohydrate diet'.

34. Please tick the level of agreement which apply to your answers OR tick if you do not know. **Click 'view as separate questions instead' above**, if you are using mobile phone.

	✱ *Required*							
	Strongly disagree	Disagree	Slightly disagree	Neutral	Slightly agree	Agree	Strongly agree	Do not know
By following a Low Carb Diet, I can keep my weight in a healthy range	○	○	○	○	○	○	○	○
If I followed a Low Carb Diet, I could become at risk of certain vitamins & minerals deficiencies	○	○	○	○	○	○	○	○
Following a Low Carb Diet could reduce my risk of developing chronic diseases in later life (i.e. diabetes, heart diseases, cancers)	○	○	○	○	○	○	○	○
A Low Carb Diet may cause side-effects such as tiredness, bad breath, constipation, mood instability	○	○	○	○	○	○	○	○
A Low Carb Diet is a good strategy for weight management	○	○	○	○	○	○	○	○
Getting sufficient vitamins and minerals from foods is essential for health	○	○	○	○	○	○	○	○
Having low risk of chronic diseases can make people live longer	○	○	○	○	○	○	○	○
If I follow(ed) a Low Carb Diet, I can (could) tolerate its side-effects such as tiredness, bad breath, constipation, and headache	○	○	○	○	○	○	○	○

35. Please tick the level of agreement which apply to your answers OR tick if you do not know. (continued)

	✱ Required							
	Strongly disagree	Disagree	Slightly disagree	Neutral	Slightly agree	Agree	Strongly agree	Do not know
My family thinks I should follow a Low Carb Diet	○	○	○	○	○	○	○	○
My friends/colleagues (would) support me to follow a Low Carb Diet	○	○	○	○	○	○	○	○
My doctor would approve of my Low Carb Diet plan	○	○	○	○	○	○	○	○
Best-selling books about Low Carb Diets helped me decided to go low carb	○	○	○	○	○	○	○	○
Information about a Low Carb Diet on the internet encourages me to follow my diet	○	○	○	○	○	○	○	○
What my family think I should do with my diet matters to me	○	○	○	○	○	○	○	○
My friends/colleagues support regarding my diet matters to me	○	○	○	○	○	○	○	○
Doctor’s approval of my choice of weight management is important to me	○	○	○	○	○	○	○	○
My diet plan is based on best-selling books	○	○	○	○	○	○	○	○
My diet plan relies mostly on information from the internet	○	○	○	○	○	○	○	○

36. Please tick the level of agreement which apply to your answers OR tick if you do not know. (continued)

	✱ Required							
	Strongly disagree	Disagree	Slightly disagree	Neutral	Slightly agree	Agree	Strongly agree	Do not know
People who are important to me (such as family, friends, etc.) think that I should NOT follow a Low Carb Diet	○	○	○	○	○	○	○	○
People around me (i.e. family, friends, or partner) have starchy / sweet foods in their habitual diets	○	○	○	○	○	○	○	○
Low Carb Diet products such as Atkins products are expensive and difficult to find in supermarkets	○	○	○	○	○	○	○	○
I do not have time to cook/prepare my meals	○	○	○	○	○	○	○	○
Joining in meal-time with other people who have starchy foods in their diets, (would) make(s) following a Low Carb Diet difficult	○	○	○	○	○	○	○	○
High price and lack of ready-to-eat Low Carb Diet foods make it difficult to follow a Low Carb Diet	○	○	○	○	○	○	○	○
When I do not have much time to cook/prepare my meals, it is (would be) difficult to follow a Low Carb Diet	○	○	○	○	○	○	○	○
I know what I have to do to make my diet 'Low Carb'	○	○	○	○	○	○	○	○
The decision to follow a Low Carb Diet is / would be entirely mine	○	○	○	○	○	○	○	○
I want to follow (or continue eating) a Low Carb Diet in the long term / in the future	○	○	○	○	○	○	○	○

37. Please tick the level of agreement which apply to your answers OR tick if you do not know. (continued)

	✱ Required							
	Strongly disagree	Disagree	Slightly disagree	Neutral	Slightly agree	Agree	Strongly agree	Do not know
Nutrition science focussing on carbohydrates in the diet is biased or not objective	○	○	○	○	○	○	○	○
The food industry is a reason why poor health is rising	○	○	○	○	○	○	○	○
I trust healthcare professionals and scientists working on dietary recommendations	○	○	○	○	○	○	○	○
Recently announced Britain's sugar tax makes me drink LESS SUGAR-SWEETENED soft drink/fizzy drink.	○	○	○	○	○	○	○	○
Recently announced Britain's sugar tax makes me drink MORE SUGAR-FREE soft drink/fizzy drink.	○	○	○	○	○	○	○	○

Attitude towards low-carbohydrate diets (cont)

38. **In my opinion, following a Low Carb Diet is …** (1:most harmful to 7:most beneficial) ✱ *Required*

	1	2	3	4	5	6	7	
Harmful to health	☐	☐	☐	☐	☐	☐	☐	Beneficial for health

38.a. Why? *Optional*

Section 4 - About You

39. Gender ✱ *Required*

○ Male ○ Female

40. Date of birth ✱ *Required*

Dates need to be in the format 'DD/MM/YYYY', for example 27/03/1980.

(dd/mm/yyyy)

41. Body weight

	✱ *Required*	✱ *Required*
Weight		Kilograms

42. Height

	✱ *Required*	✱ *Required*
Height		Centimetres

43. Ethnicity ✱ *Required*

- ○ White British (Scottish, English, Welsh, Northern Irish)
- ○ African British
- ○ Indian British
- ○ Other Asian British
- ○ Other Europeans
- ○ Asian
- ○ African
- ○ Latin American
- ○ American
- ○ Hispanic
- ○ Mixed ethnicity
- ○ Other

43.a. If you selected Other, please specify:

44. Annual income (your total household income if living in a family setting or your personal income if living alone or non-family setting)

- ○ No income
- ○ < £15,000
- ○ £15,001 - £30,000
- ○ £30,001 - £50,000
- ○ £50,000 – £80,000
- ○ > £80,000
- ○ Prefer not to say

45. Education: ✱ *Required*

- ○ School leaver/standard grade/GCSE
- ○ Highers/A levels
- ○ Higher education HND/HNC/NVQs
- ○ Bachelor's degree
- ○ Master degree/PhD/Postgraduates
- ○ Prefer not to say

46. Did your studies have a major focus on any of the following? ✱ *Required*

- ○ Nutrition
- ○ Dietetics
- ○ Food science
- ○ Medicine
- ○ Other Healthcare Professionals (i.e. nursing, midwifery, pharmacy, physiotherapist)
- ○ No, I did not study any of the above.

Section 4 - About You (cont)

47. Is your job (or part of your job) related to health, nutrition or physical fitness (i.e. doctors, nurses, pharmacists, dietitians, fitness trainer, cooking book writer, working in a media relating to health/nutrition)?

- ○ Yes, (please specify)
- ○ No

47.a. if YES, please specify below

[ ]

48. What kind of accommodation do you live in?

- ○ own room in flat/house share
- ○ student accommodation
- ○ own rented flat/house
- ○ own purchased house (mortgaged or owned outright)
- ○ Other

48.a. If you selected Other, please specify:

[ ]

49. Who do live with? (tick all that apply)

- ☐ Partner/husband/wife
- ☐ Children
- ☐ Parents
- ☐ Friends/Flatmates/Housemates
- ☐ Alone
- ☐ Other

49.a. If you selected Other, please specify:

[ ]

50. Are you the main grocery shopper in your household?

- ○ Yes, always
- ○ Yes, sometimes/occasionally
- ○ No

51. Are you the main cook in your household?

- ○ Yes, always
- ○ Yes, sometimes/occasionally
- ○ No

51.a. if YES, How confident at cooking are you from 1 (least) to 7 (highest)?

Please don't select more than 1 answer(s) per row.

	1	2	3	4	5	6	7	
Least	☐	☐	☐	☐	☐	☐	☐	Highest

Section 4 - About You (cont)

52. Do you regularly take any vitamin, mineral or other nutritional supplements? ✱ *Required*

- ◯ Yes
- ◯ No

52.a. if Yes, please specify which ones and dosage

	Supplements Name/Brand (e.g. Vitamin C Blackmore)	Dosage (e.g. 1000 mg/tab)	Frequency
1.			Please select
2.			Please select
3.			Please select
4.			Please select
5.			Please select
6.			Please select
7.			Please select

52.b. if Yes, please provide reason(s) why you take them

52.c. if NO, why not:

53. Do you smoke?

- ◯ Yes
- ◯ Ex-smoker
- ◯ No, I do not smoke.

53.a. if Yes, how many cigarettes per day

Please enter a number.

53.b. if Ex-smoker, how long have you stopped smoking?

54. Have you been told by your GP / doctor that you have any of the following? ✱ *Required*

Please select at least 1 answer(s).

- ☐ Type 1 Diabetes
- ☐ Type 2 Diabetes
- ☐ Other cause of diabetes
- ☐ High blood pressure
- ☐ Heart disease
- ☐ High blood cholesterol/ triglyceride
- ☐ Epilepsy
- ☐ Irritable bowel syndrome
- ☐ Inflammatory bowel syndrome
- ☐ Anaemia from iron deficiency
- ☐ Anaemia from other causes (Thalassemia, cancer, kidney disease)
- ☐ Other
- ☐ I have not been diagnosed with any disease

54.a. If you selected Other, please specify:

55. Are you allergic to wheat or gluten? ✱ *Required*

- ○ Yes
- ○ No

55.a. if YES, was this diagnosed by a GP / doctor?

- ○ Yes
- ○ No

56. How physically active are you? NB: 'raising heart rate' means unable to withhold a conversation at that intensity of physical activity. ✱ *Required*

- ○ Fairly inactive (walking only)
- ○ Moderately active (occasionally take exercise, that raise my heart rate, less than 3 times per week)
- ○ Very active (regularly take exercise, that raise my heart rate, 3 times a week or more)

57. Are you playing sports or a member of fitness/exercise class?

- ○ No
- ○ Yes

57.a. if YES, What sports you play/classes you attend

57.b. How often do you play/attend?

- ○ 1 day/week
- ○ 2 days/week
- ○ 3 days/week
- ○ 4 days/week
- ○ 5 days/week
- ○ 6 days/week
- ○ 7 days/week

57.c. How long do you play/attend for each session? in hour(s) or minutes per day
